# Predicting the outcome of chronic kidney disease by the estimated nephron number: The rationale and design of PRONEP, a prospective, multicenter, observational cohort study

**DOI:** 10.1186/1471-2369-13-11

**Published:** 2012-03-10

**Authors:** Toshiyuki Imasawa, Takashi Nakazato, Hiroo Ikehira, Hiroyuki Fujikawa, Ryo Nakajima, Takahito Ito, Yutaka Ando, Mitsuhiro Yoshimura, Masaru Nakayama, Kensei Yahata, Osamu Sasaki, Takaaki Yaomura, Ritsuko Katafuchi, Tsuyoshi Yamamura, Takehiko Kawaguchi, Motonobu Nishimura, Hiroshi Kitamura, Takashi Kenmochi, Akira Shimatsu

**Affiliations:** 1 National Hospital Organization Chiba-East Hospital, Chiba, Japan; 2 National Hospital Organization Chiba Medical Center, Chiba, Japan; 3 National Hospital Organization Osaka Medical Center, Osaka, Japan; 4 National Hospital Organization Osaka-Minami Medical Center, Osaka, Japan; 5 National Hospital Organization Kanazawa Medical Center, Kanazawa, Japan; 6 National Hospital Organization Kyusyu Medical Center, Fukuoka, Japan; 7 National Hospital Organization Kyoto Medical Center, Kyoto, Japan; 8 National Hospital Organization Nagasaki Medical Center, Nagasaki, Japan; 9 National Hospital Organization Nagoya Medical Center, Nagoya, Japan; 10 National Hospital Organization Fukuoka-Higashi Medical Center, Fukuoka, Japan; 11 National Hospital Organization Hokkaido Medical Center, Hokkaido, Japan; 12 Department of Nephrology, National Hospital Organization Chiba-East Hospital, 675, Nitona-cho, Chuoh-ku, Ciba-city Chiba 460-8712, Japan

**Keywords:** Chronic kidney disease, Nephron number, Birth weight, A prospective, Multicenter, Observational cohort study, Kidney biopsy, Glomerular density, Cortex volume

## Abstract

**Background:**

The nephron number is thought to be associated with the outcome of chronic kidney disease (CKD). If the nephron number can be estimated in the clinical setting, it could become a strong tool to predict renal outcome. This study was designed to estimate the nephron number in CKD patients and to establish a method to predict the outcome by using the estimated nephron number.

**Methods/Design:**

The hypothesis of this study is that the estimated nephron number can predict the outcome of a CKD patient. This will be a multicenter, prospective (minimum 3 and maximum 5 years follow-up) study. The subjects will comprise CKD patients aged over 14 years who have undergone a kidney biopsy. From January 2011 to March 2013, we will recruit 600 CKD patients from 10 hospitals belonging to the National Hospital Organization of Japan. The primary parameter for assessment is the composite of total mortality, renal death, cerebro-cardiovascular events, and a 50% reduction in the eGFR. The secondary parameter is the rate of eGFR decline per year. The nephron number will be estimated by the glomerular density in biopsy specimens and the renal cortex volume. This study includes one sub-cohort study to establish the equation to calculate the renal cortex volume. Enrollment will be performed at the time of the kidney biopsy, and the data will consist of a medical interview, ultrasound for measurement of the kidney size, blood or urine test, and the pathological findings of the kidney biopsy. Patients will continue to have medical consultations and receive examinations and/or treatment as usual. The data from the patients will be collected once a year after the kidney biopsy until March 2016. All data using this study are easily obtained in routine clinical practice.

**Discussion:**

This study includes the first trials to estimate the renal cortex volume and nephron number in the general clinical setting. Furthermore, this is the first prospective study to examine whether the nephron number predicts the outcome of CKD patients. The results from this study should provide powerful new tools for nephrologists in routine clinical practice.

**Trial registration:**

UMIN-Clinical Trial Registration, UMIN000004784.

## Background

Previous studies suggested that chronic kidney disease (CKD) is one of the most important risk factors for cardiovascular disease among known other risk factors, such as diabetes, hypertension, hyperlipidemia, obesity, smoking, and lifestyle [1,2]. Furthermore, the number of the dialysis patients resulting as a consequence of the progression of CKD has been constantly increasing. Subsequently, the medical costs for treatments for end-stage kidney disease (ESKD) patients have also been increasing in Japan [3]. CKD is also regarded as a major public health problem worldwide [4].

Low birth weight (LBW) is a risk factor for the progression of kidney diseases in compliance with the Barker hypothesis [5-9]. In particular, it is well-known that glomerular changes of adults who were born with a low birth weight show focal segmental glomerulosclerosis [10]. The nephron number is correlated with the birth weight [11,12]. Therefore, the reason why the LBW affects the progression of CKD can be explained by intra-glomerular hypertension, which is induced to compensate for the small number of nephrons [13-15]. These previous data suggest that estimation of the nephron number in CKD patients can be a useful tool to predict their outcomes. However, nephrologists currently do not have any tools to determine the individual nephron number of CKD patients under normal clinical situations. Previously, estimating the total glomerular number was accomplished by a combination of magnetic resonance imaging (MRI) and a biopsy to measure the cortical glomerular volume fraction and mean glomerular volume [16,17]. However, MRI is not realistic to use in routine clinical practice because of its high cost. In addition, in spite of the fact that measuring both the cortical glomerular volume fraction and mean glomerular volume are considered to be appropriate parameters to accurately estimate the total glomerular number in individual patients, these are also unrealistic, because these procedures are very time-consuming.

This PRONEP study was designed to establish a method for predicting the renal outcome based on the nephron number estimated in patients with CKD that can be easily used in routine clinical practice. This is the first prospective study to examine whether the nephron number can predict the outcomes of CKD patients. The nephron number will be estimated by the glomerular density in biopsied samples and the volume of the renal cortex, in which glomeruli exclusively exist. In addition, this study includes one sub-cohort study to develop an equation to calculate the renal cortex volume, because there is currently no method to measure it in the typical clinical setting.

## Methods and Design

### Hypothesis

**Figure 1:**
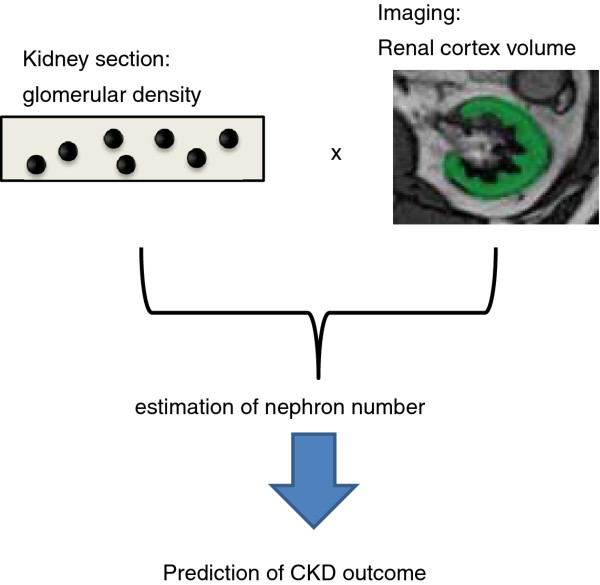
**A schematic diagram of the PRONEP study**. This study is the first prospective study to determine whether the nephron number estimated by the glomerular density in kidney biopsy specimens and the volume of the renal cortex can predict the outcome of a CKD patient.

The nephron number, which is estimated from the glomerular density in kidney biopsy specimens and the volume of the renal cortex, can predict the outcome of a CKD patient (Figure 1).

### Study design

In a multicenter, prospective (minimum 3 and maximum 5 years follow-up) study, approximately 600 patients with CKD who undergo a kidney biopsy for their diagnosis and will be enrolled from January 2011 to March 2013. These CKD patients will be recruited from 10 hospitals belonging to the National Hospital Organization of Japan. The hospitals participating in this study are spread throughout Japan (Hokkaido, Kanazawa, Chiba, Nagoya, Kyoto, Osaka, Fukuoka, and Nagasaki prefectures). This protocol was submitted to the UMIN-Clinical Trial Registration on January 1, 2011, and its unique trial number is UMIN000004784.

### Study participants

Participants will be eligible for inclusion if they (1) have CKD according to the K/DOQI CKD Guidelines [18], (2) are undergoing a needle biopsy of the kidney, (3) sign the acceptance letter for participating in this study, (4) are over 14 years old, (5) and their guardian has also signed the letter if they are under 20 years old. Participants will be excluded for any of the following reasons: (1) severe laterality in kidney size or function, for example, unilateral kidney, severe unilateral kidney atrophy, functionally unilateral kidney, and so on, (2) have had cancer, but the patients are eligible if they are free from the cancer for more than 1 year before the kidney biopsy.

In this study, the estimated glomerular filtration rate (GFR) will be calculated using the following formula, which was developed for Japanese subjects [19]:

$$\text{eGFR}\left(\text{mL}/\min/1.73{\text{m}}^{2}\right)=194\times \text{Ag}{\text{e}}^{-0.287}\times \text{Cr}{\text{e}}^{-1.094}\left(\times 0.739\;\;\text{infemals}\right)$$

In this study, all attending CKD patients will undergo a needle biopsy of the kidney for their diagnosis. Therefore, although this protocol determines the exclusion criteria by the patients' CKD stage, most of the attending patients should not be in stage 4 or 5.

### Data collection at the enrollment

**Figure 2:**
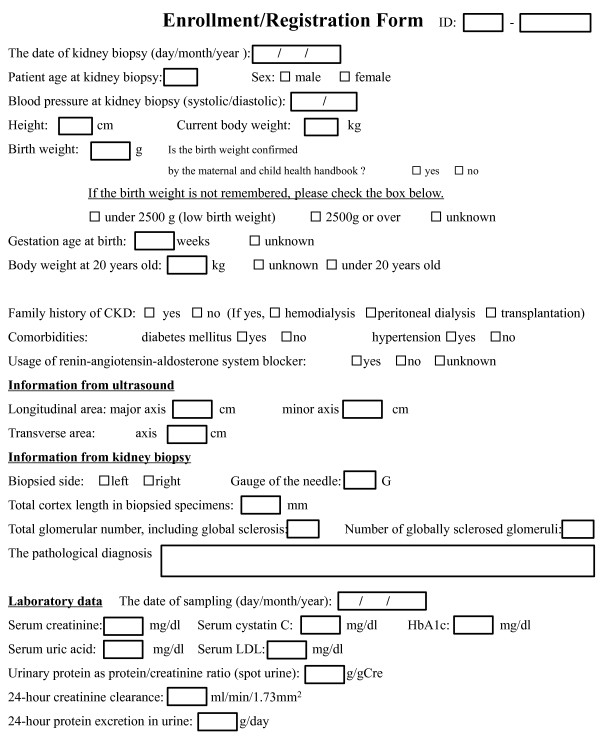
**Registration form at enrollment**. The original Japanese form was translated to English.

Enrollment should be performed at the time of the kidney biopsy after informed consent, followed by permission to participate in this study, is obtained. All patients will undergo: 1. a medical interview, 2. an ultrasound to measure the kidney size, 3. blood or urine tests, 4. a kidney biopsy to investigate the pathological findings data at the enrollment is shown in Figure 2. These examinations are performed routinely in the typical clinical setting for CKD patients receiving a kidney biopsy. In other words, no new or special examinations are included in this study.

In the medical interview, the question about birth weight may be difficult to answer, especially for elderly patients. If the subjects cannot answer the question about their birth weight, the question will be changed to whether their birth weight was normal (equal to or over 2500 g) or not, because LBW is defined as a newborn birth weight of less than 2500 g by the World Health Organization.

### Clinical follow-up and data collection

Patients will continue to have medical consultations and will receive examinations and/or treatment as usual. We will not direct physicians to prescribe any preferred drug, examination, or to give any specific medical advice. We will also not ban the physicians from prescribing any particular drugs.

**Figure 3:**
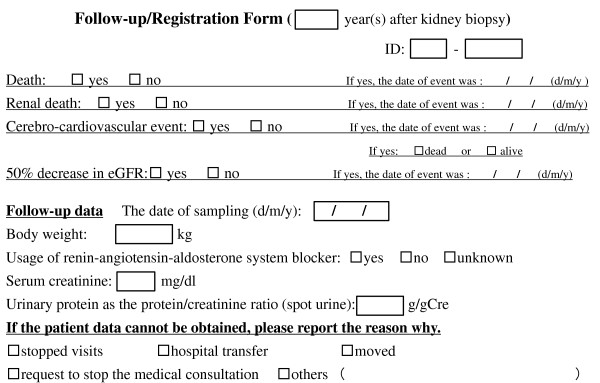
**Registration form to report follow-up data**. The original Japanese form was translated to English.

The data for patients will be collected once a year after the kidney biopsy, and will be followed until March 2016. We will permit the data collected during the antero-posterior three months of the day of the kidney biopsy to be analyzed as well. The data collection plan is shown in Figure 3.

In this study, a cerebro-cardiovascular event was defined as an acute myocardial infarction, angina pectoris, and cerebro-vascular diseases. In addition, in order for a myocardial infarction to be reported in this study, it has to fulfill at least two of the following: (1) chest symptoms, (2) ECG changes, (3) elevated cardiac enzymes. Angina is defined as the presence of ECG abnormalities with chest symptoms and the need for catheter or surgical treatment. Furthermore, a cerebro-vascular event is defined as cases with neuropathy lasting more than 24 hours continuously and with proof of a causative lesion by CT scan or MRI (TIA and asymptomatic small infarction are not included). If no data is tracked during the follow-up, the reason will be recorded (stopped visiting the hospital, transferred to another hospital, moved to a new house, offered to stop the medical consultation, and so on).

### Parameters for the assessment

The primary parameter that will be assessed is the composite of the total mortality, renal death (starting maintenance hemodialysis and peritoneal dialysis, kidney transplantation), cerebro-cardiovascular events (ischemic heart disease, cerebral hemorrhage, cerebral infarction), and a 50% reduction in the eGFR. The secondary parameter is the rate of eGFR decline per year.

### Rationale for the number of patients

This study aims to predict the outcome of chronic kidney disease using the estimated nephron number. We assumed that the time from entry to event will be independently and exponentially distributed. The planned sample size was based on a two-tailed log-rank test with the significance level set to 0.05, and the power level set at 0.80. The ratio of low birth weight infants per total newborns in Japan was reported to be 0.086 in 1960 and 0.097 in 2007. During this period, the rate has been consistently increasing http://www.mhlw.go.jp/english/database/db-hw/vs01.html. The rate of LBW may be higher in the CKD patients than in normal subjects. We assumed that the rate of LBW would be 0.095 in the CKD patients in this study. This study is planned with an accrual interval of 2 years, and additional follow-up after the accrual interval of 3 years. The event rates were assumed to be 0.1 in the normal birth weight group and 0.2 in the low birth weight group by several studies [8,20,21]. The required sample size was therefore calculated to be 50 for the low birth weight group and 476 for the normal birth weight group [22]. Assuming that 10% of subjects would withdraw, we used the simple number of 600 as the target number of patients to recruit for this study.

### Measurement of the kidney size by ultrasound imaging

**Figure 4:**
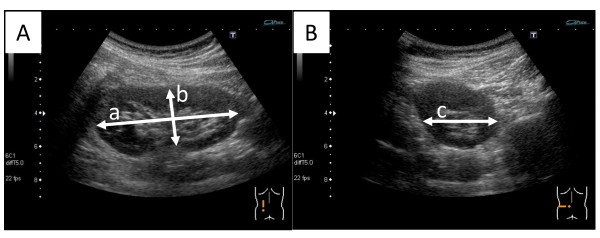
**Measurement of the kidney size by ultrasound**. (**A**) We first select an image where the renal hilum is clearly observed and the area of longitudinal plane is at the maximum. In this image, the major axis (line "a") and minor axis (line "b") are measured. The line "b" should be vertical to the major axis and pass through the center of the renal hilum. (**B**) Next, we select the maximum transverse image, which is almost a circle, and passes through the center of the renal hilum. In this image, the transverse axis (line "c") is measured.

We will record the major and the minor axis of the longitudinal plane, and the diameter of the transverse plane of the kidney by ultrasound examination as shown in Figure 4. Only the size of the biopsied site will be registered at enrollment.

### Information from kidney biopsy specimens

**Figure 5:**
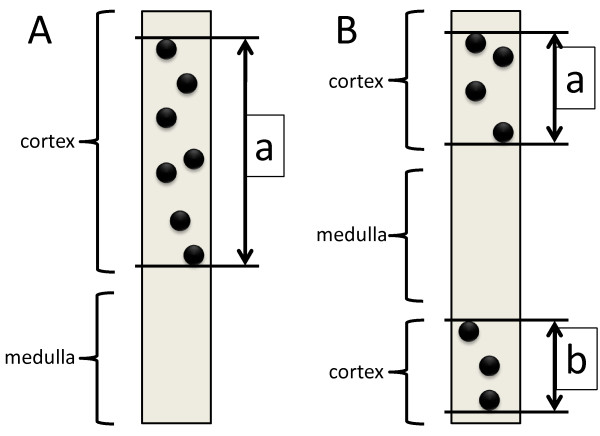
**Definition of the length of the cortex in this study**. (**A**) The length of cortex is measured from the outermost glomerulus to the innermost glomerulus. (**B**) When the medulla is sandwiched between cortices, the total of two lengths between the outermost glomerulus to the innermost glomerulus is considered the length of the cortex in the section.

As a general rule, sections stained by PAS or PAM-HE will be used for the measurement of the glomerular number and the length of the cortex (Figure 5). At the time of the measurement, squared grids will be set in an ocular lens, and thereafter, the length of the cortex will be measured. In the formal definition, the cortex and medulla are separated by the arcuate artery of the kidney. However, it is difficult to clearly discriminate between the cortex and medulla because of the frequent lack of the arcuate artery in the specimen. Therefore, we define the length of the cortex in the kidney biopsy specimen as shown in Figure 5. The needle gage number is also recorded, so that we will know the width of the specimen. The gross square area of the cortex in the specimens can be calculated by the total length of the cortex and the width. The total glomerular number and the number of glomeruli with global sclerosis will also be recorded. If the section has only one glomerulus, the section is ignored. If the total glomerular number in all sections obtained is under 8, such cases will be considered insufficient for the analysis. In line with the usual clinical procedure, the pathological diagnosis will be performed and registered.

### Estimation of the total nephron number in a kidney

Based on previous studies, we will first assume that there is 31% volume shrinkage in the paraffin-embedded specimens due to fixation in formalin [17,23,24]. Therefore, we will take the quotient of the actual measured values of the length by $0.883\;\left(=\sqrt[{3}]{1-0.31}\right)$ for the calculation below. Next, the volume of the glomerulus is thought to shrink 43% in the biopsied specimens because of the loss of arterial pressure and because of paraffin embedding after fixation in formalin [16,17,23,24]. Therefore, the actual axis of the glomerulus is calculated by dividing the measurement axis by $0.829\left(=\sqrt[{3}]{1-0.43}\right)$, and the value is used for the numerical formula below.

The total number of glomeruli is estimated as follows:

The formula of the spherical volume can be derived using integral calculus, i.e. disk integration to sum the volumes $\text{V}\;\text{ = }\;\int_{-r}^{r}\pi \left(r^{2}-x^{2}\right)dx=\frac{4}{3}\pi r^{3}$, where r is radius of the sphere, and π is the constant pi.

**Figure 6:**
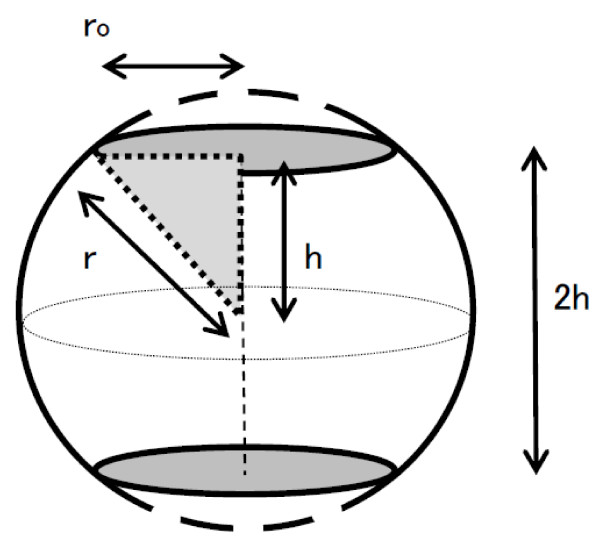
**A hypothetical sphere of a glomerulus**.

Similarly, the average area of the observed glomeruli on one section in the biopsied specimen is estimated as follows:

We assume that glomeruli less than 5 micrometers in diameter cannot be counted. A sphere 2r in diameter is cut off at both ends in parallel so that the diameter of a section may be set to 2r_o_(5 μm). We observe only circles parallel to the section of this solid, whose height is defined as 2 h. Considering a 2 h-high cylinder whose volume is equal to this solid, the cylindrical base area is equal to the average cross-sectional area of this solid cut at random parallel to the base of the solid (Figure 6).

$${\text{A}}_{\text{h}}\times 2\text{h = }\int_{-\text{h}}^{\text{h}}\pi \left({\text{r}}^{2}\text{ - }\;{\text{x}}^{2}\right)\text{dx}$$

where A_h _is the average rea of the observed circle. Hence, A_h _is estimated by:

$${\text{A}}_{\text{h}}=\frac{1}{2\text{h}}\int_{-\text{h}}^{\text{h}}\pi \left({\text{r}}^{2}\text{ - }{\text{x}}^{2}\right)\text{dx = }\frac{\pi }{2\text{h}}{\left[{\text{r}}^{2}\text{x - }\frac{{\text{x}}^{\text{3}}}{3}\right]}_{-\text{h}}^{\text{h}}=\frac{\pi }{2\text{h}}\left(2r^{2}\text{h - }\frac{2}{3}{\text{h}}^{3}\right)=\pi \left({\text{r}}^{2}-\frac{{\text{h}}^{2}}{3}\right)$$

Using the Pythagorean theorem, $\text{h}=\sqrt{{\text{r}}^{2}-{\text{r}}_{\text{o}}{}^{2}}$

Where r is the glomerular radius and r_o _is the cut-off value of the observed glomerular radius (Figure 6).

Substituting h with a function of r and r_o _gives:

$${\text{A}}_{\text{h}}=\pi \left({\text{r}}^{2}-\frac{\left({\text{r}}^{\text{2}}-{\text{r}}_{\text{o}}^{2}\right)}{3}\right)=\pi \left(\frac{2}{3}{\text{r}}^{2}+\frac{1}{3}{\text{r}}_{\text{o}}^{2}\right)$$

A_cortex_s _(Area of the cortex of the section in a biopsied specimen) is calculated as:

$${\text{A}}_{\text{cortex\_s}}={\text{L}}_{\text{a + b}}\times {\text{d}}_{\text{b}}$$

Where d_b _is the internal diameter of the biopsy needle and L_a+b _is "a + b" in Figure 5 for the total cortex length in biopsied specimens.

VF _glom/cortex _(Volume fraction of glomeruli/cortex) is equal to AF_glom/cortex _(Area fraction of glomeruli/cortex on the section).

$$\text{V}{\text{F}}_{\text{glom}/\text{cortex}}=\text{A}{\text{F}}_{\text{glom}/\text{cortex}}=\frac{{\text{N}}_{\text{s}}\times {\text{A}}_{\text{h}}}{{\text{A}}_{\text{cortex - b}}}$$

Where N_s _is the number of observed glomeruli in a biopsied specimen.

Finally, the total number of glomeruli (N_total_) was calculated as:

$${\text{N}}_{\text{total}}\;=\;\frac{\text{Total}\;\text{glomerular}\;\text{volume}}{\text{one}\;\text{average}\;\text{glomerular}\;\text{volume}\;}\;=\;\frac{\text{V}{\text{F}}_{\text{glom/cortex}}\;\times \;{\text{V}}_{\text{Cortex}}}{\frac{4}{3}\;\pi {\text{r}}^{3}}$$

Where V_cortex _is the volume of the cortex estimated by MRI.

In this study, the value of r will be decided after calculating the means of approximately 5% of the attending patients by random sampling. Therefore, "r" is not a variable in the final calculation.

### Statistical analysis

All data are expressed as the means (SD). The primary parameter for assessment is the composite of the total mortality, renal death, cerebro-cardiovascular events, and a 50% reduction in the eGFR. We will use a Cox proportional hazard model for the analysis. We will check for confounders, interactions and multicollinearity among the independent variables. The final models will be adjusted by all significant variables, as well as confounders and other baseline covariables judged to have clinical importance. The secondary parameter is the rate of eGFR decline per year. A multivariate regression analysis will be used for the analysis. The significance level on both sides in the hypothesis testing will be set at 0.05. The analyses will be performed using the SPSS statistics software program version 19.0. (IBM Corp.)

### Sub-cohort study to establish an equation for the renal cortex volume

Some of the participating patients will undergo MRI to measure the cortex volume of their kidney, because MRI is the safest procedure and has highest resolution to separate the cortex from the medulla, as reported in other studies [16,17]. However, it is unrealistic to perform MRI for all CKD patients because of its high cost. Therefore, we will attempt to establish the equation to calculate an approximate value of the kidney cortex volume measured by MRI.

### Measurement of the cortex volume by MRI

While MR imaging sometimes does not provide sufficient contrast for the soft tissues, in many cases, it is possible to distinguish the cortex and the pith of the kidney by tomography. Therefore, we will image the entire kidney with axial MRI, and calculate the renal cortical volume by multiplying the slice thickness and the slice number of the kidney which we measured on each axis tomogram. For this purpose, we found that an MR image that provided the best contrast between the cortex and medulla of the kidney was obtained using the following sequence: 2D Turbo FLASH TR = 1570 ms, TE = 2.74 ms, TI = 1000 ms, Flip Angle = 15°, Fat sat (-). We will manually draw the ROI in the kidney, and the regional choice in the axial image of the kidney will be determined by specialized radiological technologists (with AZE virtual Place FUJIN workstation, AZE Co. Ltd., Tokyo).

The calculations used for the renal cortical and whole kidney volume are as follows:

Wholekidneyvolume= ∑Areaofthekidneyineachslice×slicethickness(sliceinterval)

Renalcortexvolume= ∑Areaofthecortexineachslice×slicethickness(sliceinterval)

## Discussion

Chronic kidney disease is regarded as public health problem throughout the world [4]. In this study, we will mostly focus on the importance of the nephron number as a predictor of the outcome of CKD patients. The nephron numbers vary widely among individuals [25,26]. One report described that the range is from 227,327 to 1,825,380 per kidney, which is an 8-fold difference [12]. Compensatory adaptation to a reduced nephron number results in an increased single nephron GFR and hyperfiltration. This mechanism was described as the hyperfiltration theory by BM Brenner [14,27]. In fact, it was shown that nephron number is associated with the progression of CKD [25]. Furthermore, many reports indicate that low birth weight is a risk factor for the progression of CKD [6-9,13], which further emphasizes the importance of the nephron number, because the nephron number is positively correlated with the birth weight [11,12]. Therefore, we hypothesized that the individual nephron number would be useful for predicting the outcome of CKD.

As mentioned above, although it is likely that estimating the nephron number will be useful to predict the outcomes of CKD patients, it is currently not possible to determine the nephron number in living subjects. It was reported that the glomerular density is correlated with the prognosis of IgA nephropathy [28]. Although the glomerular density probably correlates with the nephron number, the individual renal cortex volume should largely affect the nephron number. In addition, all of the previous clinical studies concerned with birth weight or nephron number were retrospective. The purpose of this study is to examine whether the estimated nephron number can predict the outcome of CKD patients. It will be the first prospective study of this type ever to be performed.

For this purpose, 3 obstacles will have to be overcome. First, it is not possible to measure the renal cortex volume in the normal clinical setting. To develop a method for estimating this parameter, a one sub-cohort study is being performed. MRI can differentiate the cortex from the medulla because of its high resolution. Other studies also evaluated the total nephron number of dog kidneys or human donor kidneys by the cortex volume measured by MRI [16,17]. However, we cannot perform MRI for all CKD patients because it is too expensive and not always available. For this purpose, we will first accurately measure the renal cortex volume of some of the participating patients by MRI. Next, we will establish a method to calculate an approximate value of the renal cortex volume measured by MRI. The estimating equation may consist of the kidney size measured by ultrasound, patient sex, eGFR, birth weight, present body weight, and so on. This sub-cohort study will provide us with a method for determining the cortex volume in the general clinical setting. As a second step, we will need to estimate the nephron number while the subjects are still alive. Although measurements of the cortical glomerular volume fraction and mean glomerular volume in individuals are most accurate for calculating the glomerular density, such analyses are unfortunately very time-consuming and therefore a difficult to routinely perform in general clinical situations. Therefore, in this study, the total nephron number will be estimated by performing simpler analyses of biopsied specimens as described above. As the final step, the capacity of the estimated nephron number to predict the outcome of CKD patients will be assessed. The primary parameter for assessment is a composite of the total mortality, renal death, cerebro-cardiovascular events, and 50% reduction in the eGFR. The secondary parameter is the rate of eGFR decline per year. All of these parameters affect the quality of life and survival of CKD patients.

In this study, we aim to establish a method to predict the outcome of CKD patients at the time of their kidney biopsy. Therefore, the data collected during the follow-up period will be restricted (Figure 3). Because body weight may affect the serum creatinine level, we will include this data. There is a possibility that we may also analyze the impact of the urinary protein level and usage of renin-angiotensin-aldosterone system blockers, because both are major effectors of the prognosis of CKD patients [29-31]. Therefore, these data will also be collected. On the other hand, we also require a lot of data at enrollment (Figure 2), all of which will be variables of the equation used to calculate the renal cortex volume or predict the CKD outcomes. We plan to develop an equation that can be used for all CKD patients. However, nephrologists will likely need different equations for each disease, for example, diabetic nephropathy, benign nephrosclerosis, and IgA nephropathy. Therefore, we will further analyze the data by individual diseases.

This study includes the first trials to estimate the renal cortex volume and nephron number in the general clinical setting. In addition, this study is the first prospective study to examine whether the nephron number can predict the outcome of CKD patients. The results from this study should provide powerful new tools for nephrologists in routine clinical practice.

### Ethical approval

This study is being conducted in accordance with the "Ethical Guidelines for Clinical Studies" (Revised on December 28, 2004, the Ministry of Health, Labour and Welfare in Japan) and the "Ethical Guidelines for Epidemiological Studies" (Revised on August 16, 2007, the Ministries of Education, Culture, Sports, Science and Technology/Health, Labour and Welfare in Japan). All medical professionals involved in this study must comply with these ethical standards. All subjects will provide informed consent to participate in the study at the time of the kidney biopsy.

This trial was approved by the Committee on Ethics in Human Research of National Hospital Organization Chiba-East National Hospital in December 2010 (No. 19).

## Competing interests

The authors declare that they have no competing interests.

## Authors' contributions

TI: Principal investigator; the conception, design of the study and the writing of the manuscript draft; TN: statistical expertise; HI, HF, RN: developed the methodology to be used for the imaging analysis by MRI and ultrasound; TI, YA, MY, MN, KY, OS, TY, RK, TY, TK, MN: participated in the design and co-ordination of the study; HK: developed the methodology to be used for the pathological analysis; TK, AS; critical review of the manuscript. All authors read and approved the final manuscript

## Pre-publication history

The pre-publication history for this paper can be accessed here:

http://www.biomedcentral.com/1471-2369/13/11/prepub
